# Psychological impact of being a caregiver to children with infantile neuroaxonal dystrophy

**DOI:** 10.1055/s-0046-1825515

**Published:** 2026-07-21

**Authors:** Danilo Assis Pereira, Flávia Mariana Borges Montalvão Marques, Isabella Allegretti, Elaine Henna

**Affiliations:** 1 Pontifícia Universidade Católica de São Paulo, Faculdade de Ciências Médicas e da Saúde, Departamento de Reprodução Humana e da Infância, Sorocaba SP, Brazil.; 2 Pontifícia Universidade Católica de São Paulo, Faculdade de Ciências Médicas e da Saúde, Departamento de Saúde Mental, Sorocaba SP, Brazil.

**Keywords:** Caregiver Burden, Depression, Dysthymic Disorder, Quality of Life

## Abstract

**Background:**

Caring for children with infantile neuroaxonal dystrophy (INAD), a rare and progressive degenerative disease, can compromise mental health and consequently affect the quality of care given; however, there are no studies addressing this issue.

**Objective:**

To study mental health, focusing on anxiety, depression, hopelessness, quality of life, and sleep patterns among Brazilian caregivers of children with INAD, identifying associations among the variables.

**Methods:**

We enrolled 20 caregivers of children with INAD in a cross-sectional study using the following self-report questionnaires: State-Trait Anxiety Inventory, Patient Health Questionnaire-9, Beck Hopelessness Inventory, World Health Organization Quality of Life, Pittsburgh Sleep Quality Index, and Epworth Sleepiness Scale. We calculated the prevalence of psychological symptoms, and for the analyses of correlations among anxiety, depression, hopelessness, life quality, sleep quality, sociodemographic, and INAD symptoms, a Pearson's correlation test was applied.

**Results:**

Caregivers exhibited high levels of mental distress: 95% were depressed, 75% anxious, and all reported some degree of hopelessness. The quality-of-life scores were lowest in the psychological domain, and poor sleep quality was predominant. The correlation analysis uncovered that onset of physical decline in children appeared to be the most pervasive factor associated with mental suffering within our sample.

**Conclusion:**

Providing care for children with INAD is associated with severe psychological challenges, particularly in the early stages of the disease. These findings highlight the importance of early psychological screening and structured psychosocial interventions to support caregiver's wellbeing and enhance caregiving capacity

## INTRODUCTION

Caring for children with chronic and disabling conditions, such as structural epilepsy, neurodevelopmental disorder or cerebral palsy is often associated with elevated levels of mental distress, including anxiety, depression, and heightened parenting stress, which can adversely affect caregivers' quality of life (QoL). 1 2 3 Likely, parenting children with infantile neuroaxonal dystrophy (INAD) can involve similar psychological stress. Infantile neuroaxonal dystrophy is a rare, autosomal recessive and progressive neurodegenerative disorder, classified within the spectrum of PLA2G6-associated neurodegeneration (PLAN). 4

Infantile Neuroaxonal Dystrophy typically manifests between 6 and 36 months of age and progresses rapidly, causing severe motor and cognitive regression, visual and speech impairments, and often premature mortality. 4 Such natural evolution requires specialized medical support and training for parents, which can be overwhelming and cause emotional suffering, particularly in low-to-middle-income countries, where resources and support systems may be limited. Considering that poor parenting directly affects children's wellbeing, increasing their difficulties, with distressed caregivers less able to meet the psychological and physical needs of their children, 5 it is necessary to understand the emotional condition of parents. Despite the devastating nature of INAD and the likely toll it takes on caregivers, there is a notable gap in the literature regarding the mental health of this population. To our knowledge, no prior studies have specifically addressed the psychological outcomes of INAD caregivers. Therefore, we aimed to evaluate depression, anxiety, hopelessness, QoL, and sleep disorders in a sample of Brazilian caregivers of INAD patients. We also intend to explore possible associations between emotional distress and the other variables.

## METHODS

### Study design and participants

We conducted a cross-sectional online study among 20 primary caregivers (aged ≥ 18 years) of children diagnosed with INAD (confirmed or clinically compatible diagnosis). They composed a convenience sample, recruited through a national family nongovernmental organization support for INAD, and all caregivers agreed to participate.

### Data collection

All caregivers answered an online questionnaire composed of seven sections. The first section consisted of data of caregivers' demographic characteristics and clinical neuropsychomotor symptoms of INAD children. The subsequent sections included self-report instruments to assess anxiety, depression, hopelessness, life quality, and sleep patterns.

#### *Anxiety*

We investigated anxiety as state and trait using the State-Trait Anxiety Inventory (STAI). The STAI comprises two subscales to measure state and trait anxiety. State anxiety reflects a temporary emotional condition, while trait anxiety indicates a general tendency to perceive situations as threatening. Each subscale consists of 20 items rated on a 4-point Likert scale. Scores ≥ 41 indicate significant anxiety levels in each subscale. 6

#### *Depression*

Depression was screened by the Patient Health Questionnaire-9 (PHQ-9), designed for screening, diagnosing and evaluating depression severity. Scores range from o to 27, and scores ≥ 9 indicate clinical depression. 7

#### *Hopelessness*

Hopelessness was assessed by the Beck Hopelessness Scale (BHS), which encompasses 20 questions assessing 3 major aspects of hopelessness (feelings about the future, loss of motivation, and expectations). Scores range from 0 to 20, with higher scores indicating greater hopelessness. Scores ≥ 9 indicate significant hopelessness. 8

#### *Quality of life (QoL* )

Life quality was evaluated by the World Health Organization Quality of Life - Brief (WHOQOL-BREF), it comprises 2 items for general quality of life, and 24 questions assessing 4 domains of quality of life: physical health, psychological health, social relationships, and environment. The raw scores are normalized and transformed into a 0-to-100 scale. A cutoff of 60 discriminated correctly between life satisfaction and dissatisfaction. 9

#### *Sleep patterns*

Sleep was evaluated by the Epworth Sleepiness Scale (ESS), 10 which measures the likelihood of falling asleep during eight daily situations; and by the Pittsburgh Sleep Quality Index (PSQI): a 19-item questionnaire scored on a 4-point Likert scale that measures sleep quality in the previous month. Scores ranging from 0 to 21, with higher scores reflecting poorer sleep quality. Scores ≥ 5 indicates poor sleep quality. 11

### Statistical analysis

The data were analyzed using IBM SPSS Statistics for Windows (IBM Corp.), version 25.0. Categorical variables were expressed as absolute numbers and percentages (%), while continuous variables were expressed as mean ± standard deviation. Descriptive statistics (means, standard deviations, frequencies) were used to characterize the sample.

Aiming to analyze correlation depression, anxiety, hopelessness, sleep quality and life quality with the sociodemographic and children's characteristics, a Spearman's correlation test was applied. The level of significance was set at 5%. The study was approved by Ethics Committee of Pontifícia Universidade Católica de São Paulo (# 65887222.0.0000.5373).

## RESULTS

### Characteristics of children with INAD

The mean age of the children with INAD was 8.2 ± 4.1 years. A genetic diagnosis confirming a PLA2G6 mutation was present in 61.9% of the children. The mean age at symptom onset was of 15.5 ± 5.1 months; the mean age at motor regression onset was of 17.1 ± 8.5 months; and the reduction in social interaction started at the mean age of 37.2 ± 23.6 months. Motor decline was observed in 95% of the children. The most frequent symptoms observed were spasticity (76%), visual impairment (61.9%), epilepsy (42.8%), and dystonia (42.8%). All children were alive during data collection.

### Caregivers' characteristics

A total of 20 caregivers, with a mean age of 37.4 ± 8.8 years, took part in the present study; 17 (85%) were mothers, and their mean age at childbirth was of 29.9 ± 7.5 years. Household income distribution varied, ranging from up to two, two-to-three, and more than three Brazilian minimum wages ( Table 1 ). Table 2 describes the psychological caregiver's distress. Most caregivers exhibited clinically significant anxiety and depressive symptoms, and all caregivers presented hopelessness. Quality of life was also affected, with the psychological domain being the most impacted. Additionally, the sample showed poor sleep patterns and excessive daytime sleepiness.

**Table 1:** Sociodemographic profile of the caregivers

Variable	Caregivers (n = 20)
**Mean age (years)**	37.4 ± 8.8
**Primary caregiver**	**n (%)**
Mother	17 (85)
Father	3 (15)
Total	20 (100)
**Education**	n (%)
Junior high	3 (15)
High School	4 (20)
Incomplete college	4 (20)
College	9 (45)
Total	20 (100)
**Income**	**n (%)**
< up to BRL R$ 3,039.00	5 (25)
BRL R$ 3,040.00	5 (25)
> BRL R$ 3,041.00	10 (50)
Total	20 (100)
**Private health care**	**n (%)**
Yes	17 (85)
No	3 (15)
Total	20 (100)

**Table 2:** Descriptive statistics of psychological variables assessed in caregivers

	INAD caregivers	Brazilian* cutoff
Mean anxiety-trait (STAI) ^*^	46 ± 6.8	42
**Frequencies**	**n (%)**	**-**
no/low	5 (25)	–
moderate	15 (75)	–
high	–	–
Mean anxiety-state (STAI ^*^ )	44.5 ± 5.7	42
**Frequencies**	**% (n)**	**-**
no/low	30 (6)	–
moderate	70 (14)	–
high	–	–
Mean depression (PHQ-9 ^*^ )	12.1 ± 6.5	5
**Frequencies**	**n (%)**	**-**
no	1 (5)	–
mild	6 (30)	–
moderate	7 (35)	–
moderately severe	3 (15)	–
severe	3 (15)	–
Mean hopelessness (BHI ^**^ )	10.7 ± 2.9	9
**Frequencies**	**n (%)**	**-**
no	–	–
mild	2 (10)	–
moderate	16 (80)	–
severe	2 (10)	–
	**Mean ± SD**	**Cutoff**
Overall quality of life (WHQOL ^*^ )	58.1 ± 9.9	60
**Physical domain**	63.5 ± 12.6	60
**Psychological domain**	47.5 ± 18.3	60
**Social relationship domain**	53.4 ± 20.6	60
**Environment domain**	56.3 ± 12.8	60
Diurnal somnolence (ESS ^*^ )	9.1 ± 5.2	10
Sleep quality (PSQI ^***^ )	7.6 ± 4.2	5

Abbreviations: BHS, Beck Hopelessness Scale; ESS, Epworth Sleepiness Scale; PHQ-9, Patient Health Questionnaire; PSQI, Pittsburgh Sleep Quality Index; SD, standard deviation; STAI, State-Trait Anxiety Inventory; WHOQOL, World Health Organization Quality of Life.

Notes: *All cutoffs are based on Brazilian validation studies. Scores above the cutoffs on anxiety, depression, **hopelessness, diurnal somnolence, and ***sleep quality represent suffering. Scores below the cutoff on Qol dimensions represent poor quality of life.

### Correlations results

Anxiety was associated with dystonia ( *p* = 0.499; *p* = 0.01), reduction in social interaction ( *p* = 0.495; *p* = 0.01), and depression ( *p* = 0.451; *p* = 0.02). Depression was associated with the beginning of motor regression ( *p* = 0.396; *p* = 0.04); and hopelessness with overall QoL ( *p* = 0.842; *p* < 0.001), and spasticity ( *p* = −0.469, *p* = 0.003).

Physical domain in QoL was related to epilepsy (r = −0.429; *p* = 0.03); and sleep quality was correlated with beginning of motor regression ( *p* = 0.468; *p* = 0.01), depression ( *p* = 0.402; *p* = 0.03), and dystonia ( *p* = 0.439; *p* = 0.05).

## DISCUSSION

The present study is the first to examine the psychological burden experienced by caregivers of children with INAD, an ultra-rare neurodegenerative disorder. Our findings revealed a consistent pattern of elevated mental distress, specifically depression, anxiety, and hopelessness, as well as compromised QoL and sleep disturbances.

Providing care for individuals with chronic disorders is often associated with mental distress, 2 reduced QoL, and elevated levels of depression, anxiety, and hopelessness. 12 13 14 For example, a study involving caregivers of individuals with Lennox-Gastaut syndrome found that 61.8% experienced anxiety and 50.9% experienced depression, 14 which is considerably lower than our results. This discrepancy may be partially explained by the progressive and fatal trajectory of INAD, worsening caregivers' suffering.

Based on previous studies, 5 we expected that limited financial resources could negatively affect caregivers' QoL, but this was not observed. The association observed was between low income and high anxiety.

Being religious was related to better scores in psychological domain in QoL. Possibly, attending religious services often helps individuals connect with others and find a sense of purpose. The variables most strongly associated with the symptoms evaluated in our study were those related to INAD itself, which is in line with findings from previous research involving caregivers of individuals with chronic illnesses. 15 16 17 18

In our sample, the initial signs of the disease, as well as a few symptoms, were somehow associated with all emotional variables studied. Beginning of motor regression was associated with high anxiety, presence of depression, and compromised general QoL. Epilepsy and dystonia appeared in various combinations depending on the psychological variable examined. Emotional distress among parents of children with severe disabilities has been documented 5 14 18 ; however, Parkes et al., 3 when studying parental stress and children with cerebral palsy, found that parental stress was primarily associated with disruptive behaviors in children, regardless of their overall symptomatology. These findings underscore the multitude of factors contributing to parental stress.

Hopelessness was present in all caregivers, and associated with INAD's symptomatology, indicating a pervasive sense of pessimism and despair. Hopelessness has been linked to depression and poor parenting practices, which may, in turn, exacerbate the children's difficulties. 5 Although no studies directly assessing hopelessness were identified, qualitative research conducted by Brown et al. 14 and Wilson et al. 18 among caregivers of individuals with neurodegenerative diseases, uncovered themes of pessimism that align with the concept of hopelessness.

Conversely, it has been noted that providing caregivers with information about chronic, progressive conditions helped them to cope and improved their QoL. 5 Therefore, we suggest that having access to appropriate care—such as early diagnosis, education about disease progression and prognosis, and support from peers or professionals—might help mitigate emotional suffering among caregivers. Although not representative, two mothers from our sample (both scoring low for hopelessness, with one not experiencing depression) were actively involved in supporting other caregivers within our sample. This behavior appeared to alleviate their suffering and provided them with a sense of purpose. While we did not formally evaluate this aspect, engagement in helping others is generally associated with increased wellbeing.

The QoL of caregivers was compromised across all dimensions, but the most affected domain in QoL was the psychological domain, which can be expected, once anxiety and depression symptoms are directly related to the psychological domain.

The cross-sectional design of the study precludes us from making definitive conclusions; nonetheless, our results suggest that caring for INAD patients is associated with emotional distress and diminished QoL. In particular, the onset of physical decline in children appeared to be the most pervasive factor associated with it within our sample. To our knowledge, this is the first study examining depression, anxiety, hopelessness, and sleep patterns among caregivers of patients with INAD, and it may have some implications. First, all caregivers of patients with INAD should be screened for depression, hopelessness, anxiety, and sleep disturbances; a few individuals will require specialized care. Second, since the onset of symptoms was the most robust predictor of caregiver suffering, we recommend that all caregivers should receive education about INAD to better manage disease progression. Additionally, strategies such as meditation could be encouraged, as evidence suggests it can effectively reduce distress among caregivers of individuals with dementia. 18

The current study presents several limitations. The cross-sectional design precludes the establishment of causal relationships; the small sample size of caregivers prevents the generalizability of the findings. Furthermore, the absence of a control group comprising caregivers of children with other conditions restricts direct comparisons of distress levels. So, our results should be seen as a trend that needs confirmatory studies.

In conclusion, the present study underscores the psychological impact of caregiving for children with INAD, characterized by elevated levels of depression, anxiety, hopelessness, and significantly reduced QoL. Notably, early disease milestones, particularly motor regression and the onset of symptoms, were linked to caregiver distress. These findings highlight the critical importance of implementing early psychological screening, providing anticipatory guidance, and integrating mental health support into care protocols for caregivers of patients with INAD. Ultimately, our results emphasize that improving caregivers' wellbeing is not only vital for their own health but also has the potential to enhance the quality of care provided to affected children. Future research is needed in order to confirm our results for the development of strategies to mitigate caregivers' distress and fostering resilience in this vulnerable population.
